# Design and Rationale of the National Tunisian Registry of Percutaneous Coronary Intervention: Protocol for a Prospective Multicenter Observational Study

**DOI:** 10.2196/24595

**Published:** 2022-08-05

**Authors:** Rania Hammami, Selim Boudiche, Tlili Rami, Nejeh Ben Halima, Ahmed Jamel, Bassem Rekik, Rym Gribaa, Ben Mrad Imtinene, Salma Charfeddine, Tarek Ellouze, Amine Bahloul, Ben Slima Hédi, Jamel Langar, Habib Ben Ahmed, Zied Ibn Elhadj, Mohamed Hmam, Mohamed Aymen Ben Abdessalem, Sabri Maaoui, Sana Fennira, Laroussi Lobna, Majed Hassine, Sami Ouanes, Drissi Mohamed Faouzi, Souad Mallek, Abdallah Mahdhaoui, Dghim Meriem, Walid Jomaa, Sofien Zayed, Tawfik Kateb, Nidhal Bouchahda, Fares Azaiez, Helmi Ben Salem, Morched Marouen, Aymen Noamen, Salem Abdesselem, Denguir Hichem, Hassen Ibn Hadj Amor, Farhati Abdeljelil, Amine Amara, Karim Bejar, Ben Hamda Khaldoun, Chiheb Hamza, Mohsen Ben Jamaa, Sami Fourati, Faycal Elleuch, Zeineb Grati, Slim Chtourou, Sami Marouene, Mohamed Sahnoun, Morched Hadrich, Maalej Mohamed Abdelkader, Hatem Bouraoui, Kamel Kamoun, Moufid Hadrich, Tarek Ben Chedli, Mohamed Akrem Drissa, Hanene Charfeddine, Nizar Saadaoui, Gargouri Achraf, Siala Ahmed, Mokdad Ayari, Marsit Nabil, Sabeur Mnif, Maher Sahnoun, Helmi Kammoun, Khaled Ben Jemaa, Gharbi Mostari, Nebil Hamrouni, Maazoun Yamen, Yassine Ellouz, Zahreddine Smiri, Amine Hdiji, Jerbi Bassem, Wacef Ayadi, Amir Zouari, Chedly Abbassi, Boujelben Masmoudi Fatma, Kais Battikh, Elyes Kharrat, Imen Gtif, Milouchi Sami, Leila Bezdah, Salem Kachboura, Mohamed Faouzi Maatouk, Sondes Kraiem, Gouider Jeridi, Elyes Neffati, Samir Kammoun, Youssef Ben Ameur, Wafa Fehri, Habib Gamra, Lilia Zakhama, Faouzi Addad, Mourali Mohamed Sami, Leila Abid

**Affiliations:** 1 Department of Cardiology, Hédi Chaker Hospital Faculty of Medicine of Sfax University of Sfax Sfax Tunisia; 2 Department of Cardiology, La Rabta Hospital Faculty of Medicine of Tunis University of Tunis Tunis Tunisia; 3 Department of Cardiology, Mongi Slim Hospital Faculty of Medicine of Tunis University of Tunis Tunis Tunisia; 4 Department of Cardiology, Kairouan Hospital Faculty of Medicine of Sousse University of Sousse Kairouan Tunisia; 5 Department of Cardiology, Sahloul Hospital Faculty of Medicine of Sousse University of Sousse Sousse Tunisia; 6 Department of Cardiology, Habib Thameur Hospital Faculty of Medicine of Tunis University of Tunis Tunis Tunisia; 7 Department of Cardiology, Menzel Bourguiba Hospital Faculty of Medicine of Tunis University of Tunis Bizerte Tunisia; 8 Cardiologist, Private Sector Tunis Tunisia; 9 Department of Cardiology, Charle Nicole Hospital Faculty of Medicine of Tunis University of Tunis Tunis Tunisia; 10 Department of Cardiology, Abderrahmen Mami-Ariana Hospital Faculty of Medicine of Tunis University of Tunis Ariana Tunisia; 11 Department of Cardiology, Farhat Hached Hospital Faculty of Medicine of Sousse University of Sousse Sousse Tunisia; 12 Cardiologist, Private Sector Monastir Tunisia; 13 Department of Cardiology A, Fattouma Bourguiba University Hospital Faculty of Medicine of Monastir University of Monastir Monastir Tunisia; 14 Department of Cardiology, The Main Military Instruction Hospital of Tunis Faculty of Medicine of Tunis University of Tunis Tunis Tunisia; 15 Department of Cardiology B, Fattouma Bourguiba University Hospital Faculty of Medicine of Monastir University of Monastir Monastir Tunisia; 16 Cardiologist, Private Sector Sousse Tunisia; 17 Cardiologist, Private Sector Sfax Tunisia; 18 Department of Cardiology, Gabes Hospital Faculty of Medicine of Sfax University of Sfax Gabes Tunisia; 19 Department of Cardiology, Habib Bourguiba Hospital Faculty of Medicine of Sfax University of Sfax Medenine Tunisia; 20 Cardiologist, Private Sector Nabeul Tunisia; 21 Cardiologist, Private Sector Medenine Tunisia; 22 Cardiologist, Private Sector Bizerte Tunisia

**Keywords:** percutaneous coronary intervention, 1-year outcome, Tunisia, national, multicentric, registry, percutaneous, coronary, artery disease

## Abstract

**Background:**

Coronary artery diseases remain the leading cause of death in the world. The management of this condition has improved remarkably in the recent years owing to the development of new technical tools and multicentric registries.

**Objective:**

The aim of this study is to investigate the in-hospital and 1-year clinical outcomes of patients treated with percutaneous coronary intervention (PCI) in Tunisia.

**Methods:**

We will conduct a prospective multicentric observational study with patients older than 18 years who underwent PCI between January 31, 2020 and June 30, 2020. The primary end point is the occurrence of a major adverse cardiovascular event, defined as cardiovascular death, myocardial infarction, cerebrovascular accident, or target vessel revascularization with either repeat PCI or coronary artery bypass grafting (CABG). The secondary end points are procedural success rate, stent thrombosis, and the rate of redo PCI/CABG for in-stent restenosis.

**Results:**

In this study, the demographic profile and the general risk profile of Tunisian patients who underwent PCI and their end points will be analyzed. The complexity level of the procedures and the left main occlusion, bifurcation occlusion, and chronic total occlusion PCI will be analyzed, and immediate as well as long-term results will be determined. The National Tunisian Registry of PCI (NATURE-PCI) will be the first national multicentric registry of angioplasty in Africa. For this study, the institutional ethical committee approval was obtained (0223/2020). This trial consists of 97 cardiologists and 2498 patients who have undergone PCI with a 1-year follow-up period. Twenty-eight catheterization laboratories from both public (15 laboratories) and private (13 laboratories) sectors will enroll patients after receiving informed consent. Of the 2498 patients, 1897 (75.9%) are managed in the public sector and 601 (24.1%) are managed in the private sector. The COVID-19 pandemic started in Tunisia in March 2020; 719 patients (31.9%) were included before the COVID-19 pandemic and 1779 (60.1%) during the pandemic. The inclusion of patients has been finished, and we expect to publish the results by the end of 2022.

**Conclusions:**

This study would add data and provide a valuable opportunity for real-world clinical epidemiology and practice in the field of interventional cardiology in Tunisia with insights into the uptake of PCI in this limited-income region.

**Trial Registration:**

Clinicaltrials.gov NCT04219761; https://clinicaltrials.gov/ct2/show/NCT04219761

**International Registered Report Identifier (IRRID):**

RR1-10.2196/24595

## Introduction

Coronary artery diseases remain the leading cause of death in the world [1]; the management of this condition has improved, thanks to new technical tools and multicentric registries. Recently, in Tunisia, the number of intervention procedures has markedly increased, given the explosion of cardiovascular risk factors among Tunisians [2]. However, there is a paucity of data about the short- and long-term results of percutaneous coronary intervention (PCI) in different hospitals in Tunisia, and thus, a registry of PCI procedures was initiated. Currently, there is a need to know the PCI outcomes by using this registry data and any deficiencies in patient management to help formulate improvement strategies. We will conduct this national registry to determine the current practice of PCI at our hospital in Tunisia, including the clinical characteristics, angiographic profile, and in-hospital and 1-year clinical outcomes of patients who have undergone PCI. Furthermore, this registry could be used to determine clinicians’ adherence to the published guidelines for PCI, including the different gaps in real-world practice. The aim of this study is to report the in-hospital and 1-year clinical outcome of consecutive patients undergoing PCI. This study would also generate local data that can be compared with those in other parts of the world, which would help local health care authorities to plan PCI strategies in Tunisia [3,4].

## Methods

We will conduct a prospective multicentric observational study of all patients who underwent PCI in Tunisia between January 31 and June 30, 2020 with a 1-year follow-up. Written informed consent will be obtained from all the patients. Males and females older than 18 years, admitted in public sector as well as in private sector catheterization laboratories, and who underwent a PCI during the study period will be included in this study. Each patient will be included only once during index PCI admission. Repeat admission for PCI of other vessels will be considered during the follow-up of the patients. Data will be collected from computer medical records and captured for analysis by Dacima Consulting according to the Food and Drug Administration 21 Code of Federal Regulations part 11, Health Insurance Portability and Accountability Act, and the International Conference on Harmonization requirements. One-year follow-up data will be collected either from clinic visits or by telephone. For this study, institutional ethical committee approval was obtained (0223/2020).

Statistical analyses will be performed for the risk factors, clinical presentation, angiographic profile, PCI details, stents, medication use, and in-hospital and 1-year outcome following PCI. Baseline characteristics of the patients will be analyzed in terms of frequencies and percentages for categorical variables and means and standard deviation for continuous variables. The definitions of data variables in the case report form are based on the American College of Cardiology/American Heart Association guidelines [5,6]. Conventional risk factors, including age, gender, diabetes, hypertension, dyslipidemia, current smoking (within 1 year), and family history of coronary artery disease, will be noted. Previous conditions such as myocardial infarction, PCI, coronary artery bypass grafting (CABG), peripheral vascular disease, cerebrovascular accident or transient ischemic attack, and chronic kidney disease will be assessed. Diabetes is defined according to the guidelines of the American Diabetes Association 2020, as having a history of diabetes diagnosed and treated with medication and insulin, or fasting blood glucose of 7 mmol/L (126 mg/dL), or hemoglobin A_1c_ ≥6.5%, or signs of hyperglycemia associated with a random plasma glucose of ≥200 mg/dL (11.1 mmol/L) [7]. Hypertension is defined as having a history of hypertension diagnosed and treated with medication or blood pressure ≥140 mm Hg systolic or 90 mm Hg diastolic on at least 2 occasions [8]. Hyperlipidemia is defined as a history of dyslipidemia diagnosed or treated by a physician or total cholesterol >2 g/L. Current smoker is defined as smoking cigarettes, water pipe, cigar, or chewing tobacco within 1 year of admission. Moderate chronic kidney disease is defined as estimated glomerular filtration rate <60 mL/min/1.73 m^2^ for 3 months or more, with or without kidney damage or on dialysis. Angiographic and procedural notes were reviewed. Single vessel disease was considered present if there was more than 70% diameter stenosis on visual assessment in the left anterior descending, left circumflex, or right coronary arteries, or a major branch, or more than 50% for left main stenosis and in-stent restenosis. The stented artery, number of stents used, type of stent (bare metal stent or drug-eluting stent), procedural success, and complications were noted. Left ventricular ejection fraction was noted from echocardiography. PCI was performed according to standard clinical practice. The vascular approach as well as complications related to this route will be noted. A large hematoma will be defined as >5 cm. Significant bleeding will be defined as hemoglobin drop >5 g or required >2 packs of red blood cells for transfusion.

All patients will be followed up daily until discharge. The control of the renal function will be ensured if possible. Contrast-induced nephropathy is defined as either a 25% increase in serum creatinine from baseline or 44 μmol/L increase in the absolute value within 48-72 hours of PCI. The primary end point of this study is the occurrence of major adverse cardiovascular events, defined as cardiovascular death, any myocardial infarction, cerebrovascular accident, and target vessel revascularization with either repeat PCI or CABG. Myocardial infarction is documented by the highly sensitive troponin T rise (>14 pg/mL) with either ischemic symptoms or ST elevation/depression or new pathologic Q waves on electrocardiogram after discharge or as documented in outpatient notes. Post-PCI infarction is considered as >5 times rise in troponin T from baseline levels. Target vessel revascularization is defined as any repeat percutaneous intervention or surgical bypass of any segment of the target vessel, which was stented before. The secondary end points are (1) procedural success rate, defined as successful PCI without associated in-hospital major clinical complications; (2) stent thrombosis, defined as definite stent thrombosis occurring when clinical presentation was consistent with acute coronary syndrome and angiography examination confirmed stent occlusion or thrombus; and (3) rate of in-stent restenosis, defined as >50% angiographic restenosis on follow-up within 1 year, resulting in either repeat PCI or CABG.

## Results

**Figure 1:**
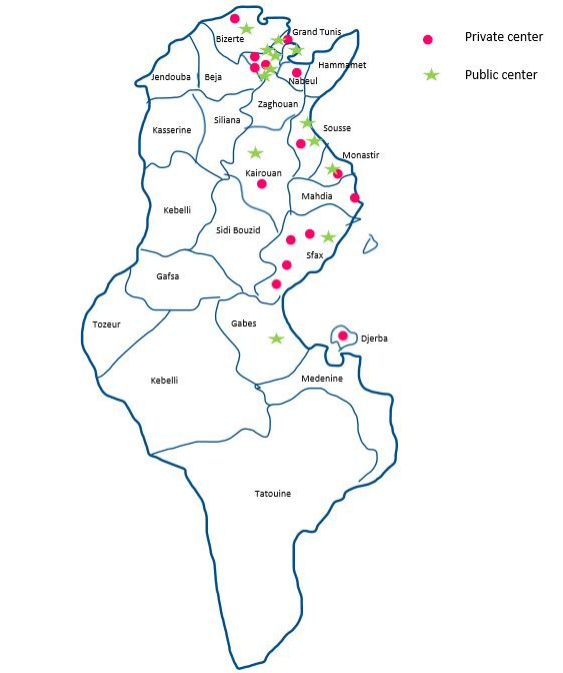
Repartition of the investigating centers in this study.

This study will enroll 97 cardiologists and 2498 patients with a 1-year follow-up period (Multimedia Appendix 1 and Multimedia Appendix 2). Twenty-eight catheterization laboratories, that is, 15 laboratories from the public sector and 13 laboratories from the private sector will enroll patients after receiving informed consent. Of the 2498 patients, 1897 (75.9%) are managed in the public sector and 601 (24.1%) are managed in the private sector (Figure 1). The COVID-19 pandemic started in Tunisia in March 2020; 719 patients (31%) were included before the COVID-19 pandemic and 1779 (60%) were included during the pandemic. The results of this study are expected to be published by the end of 2022.

## Discussion

This is the first observational registry of PCIs in Africa, which included 2498 patients. Although results from randomized controlled trials provide the highest level of evidence regarding the efficacy of interventions, they have well-recognized limitations. Randomized controlled trials may not always reflect “real-world” medical settings and often underrepresent significant portions of the community such as women and older adults [9].

Clinical registries have consequently emerged as a powerful tool to assess health care effectiveness and safety and improve quality of care, as well as to inform on the real-world impact of new interventions or medications outside the confines of randomized controlled trials. Over the last 2 decades, there has been a substantial growth in national and major regional PCI registries, predominantly in high-income countries. Numerous registries and surveys have been described in different European, Asian, and American countries, but there are only few contemporary data on the demographic characteristics and outcomes of interventional cardiology practice in low-income countries owing to concerns about costs [10]. Invasive cardiology was initiated in North Africa more than 50 years ago, with the first catheterization procedure performed in Tunis in 1968. The first coronary angiogram was performed in 1983, and the first coronary angioplasty was performed in 1989 [11]. Since its emergence as a new subspecialty, interventional cardiology has evolved quite rapidly in North Africa compared to that in other African countries apart from South Africa. The proximity of these African countries to Europe and particularly to France has helped that progression, as trainees have relatively easy access to French centers for subspecialty training.

According to a recent epidemiologic study (Tunisian Association on Study and Research on Atherosclerosis Survey), the prevalence of cardiovascular risk factors has increased widely; more than half of Tunisians have hypertension and 19% have diabetes [2]. Certainly, these conditions will affect the long-term outcomes of PCI in Tunisia, especially that the use of drug-eluting stent has many cost concerns. In Tunisia, this interventional activity is performed in 28 catheterization laboratories, located mostly in the northern and the middle-eastern region of the country. Fourteen catheterization laboratories are in the public sector. More than 100 interventional cardiologists perform at least one PCI per week.

Recently, a Tunisian national registry of myocardial infarction (FAST-MI) was set up by the Tunisian Society of Cardiology and Cardiovascular Surgery to assess the demographic and clinical characteristics, management, and hospital outcomes of patients with ST-elevation myocardial infarction (STEMI). Data for 459 consecutive patients (mean age 60.8 years, 88.5% male) with STEMI treated in 16 public hospitals (representing 72.2% of the public hospitals in Tunisia treating patients with STEMI) were collected prospectively. The most common risk factors were smoking (63.6%), hypertension (39.7%), diabetes (32%), and dyslipidemia (18.2%) [12]. The limitation of that study was the small number of patients from private hospitals. Our registry will try to project the real-world practice of interventional cardiology, both in private and public sectors. However, the COVID-19 pandemic as well as Ramadan will certainly impact the number of patients in our registry. Given the risk of health care personnel contracting infections, the activities of catheterization laboratories in different countries have dramatically decreased—reduced to nearly 70% of their normal duties.

This large contemporary longitudinal study of Tunisian PCIs will provide a unique opportunity to answer many questions. The National Tunisian Registry of PCI (NATURE-PCI) study is important in several respects. First, systematic observational and outcomes data can be generated from this registry study, which are especially valuable, given that evidence for Tunisian patients undergoing PCI is limited. Second, the follow-ups of complex procedures, especially those of the left main occlusion, chronic total occlusion, and primary PCI are changing dramatically and need to be evaluated in real-world studies. Third, the NATURE-PCI study provides a good opportunity to compare the risk of stent failure in a population with a high prevalence of cardiovascular risk factors, especially diabetes, for comparison with that of populations in other countries and to evaluate clinicians’ adherence to the guidelines of the European Society of Cardiology on myocardial revascularization.

NATURE-PCI will fill a significant gap in the dynamic landscape of interventional cardiology practice care and research. It will provide unique and necessary data on the management and outcomes of patients with coronary artery diseases who are treated invasively. This study will yield the largest contemporary longitudinal cohort of PCI in Tunisia and provide a valuable opportunity for real-world clinical epidemiology with insights into the uptake and the difficulties of PCIs. The data of this registry will be useful for considering general health care costs such as the reimbursement of drug-eluting stents in clinical settings.

## Appendix

Multimedia Appendix 1 Repartition of inclusion procedures according to months (N=2498).

Multimedia Appendix 2 Repartition of inclusion in different Tunisian cities.

## Abbreviations

CABG: coronary artery bypass grafting
NATURE-PCI: National Tunisian Registry of Percutaneous Coronary Intervention
PCI: percutaneous coronary intervention
STEMI: ST-elevation myocardial infarction
