# Supplementary figures and images for: Design and Rationale of the National Tunisian Registry of Percutaneous Coronary Intervention: Protocol for a Prospective Multicenter Observational Study
Source: JMIR Res Protoc. 2022 Aug 5;11(8):e24595. doi: 10.2196/24595 (PMC9391981; doi:10.2196/24595)

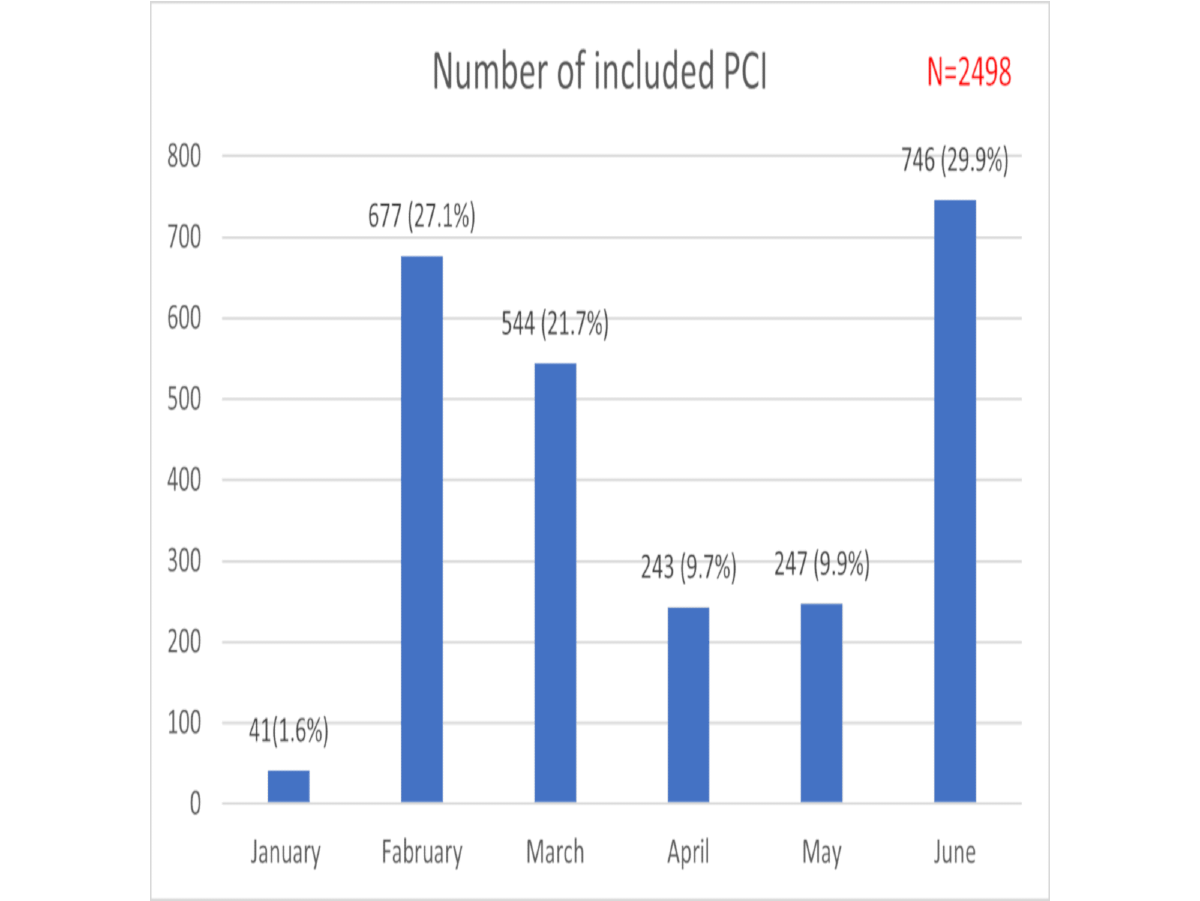

Supplement: Multimedia Appendix 1 [file resprot_v11i8e24595_app1.png]

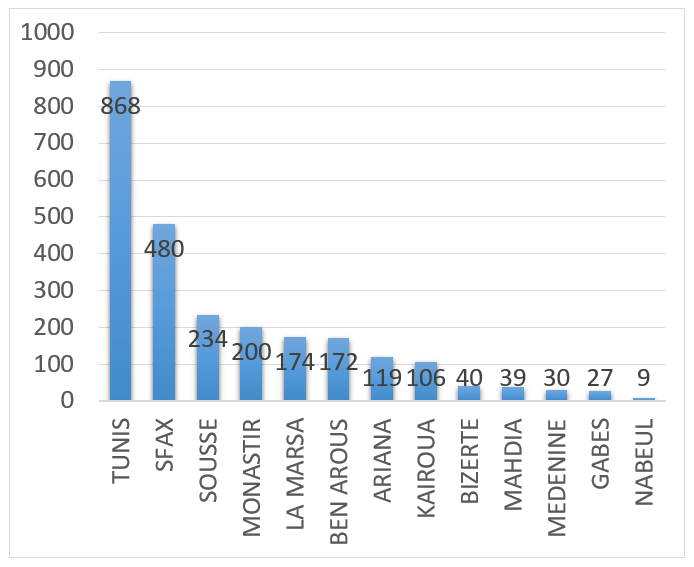

Supplement: Multimedia Appendix 2 [file resprot_v11i8e24595_app2.png]
